# The complete chloroplast genome of *Sedum tricarpum* Makino. (Crassulaceae)

**DOI:** 10.1080/23802359.2021.2005489

**Published:** 2021-12-10

**Authors:** Jing Chen, Hui Zhou, Wei Gong

**Affiliations:** a College of Life Science, Zhejiang Chinese Medical University, Hangzhou, China; b College of Life Sciences, South China Agricultural University, Guangzhou, China

**Keywords:** *Sedum tricarpum*, chloroplast genome, succulent herb, illumina sequencing

## Abstract

*Sedum tricarpum* Makino., is a perennial succulent herb, which was first discovered and established as a new species ten years ago. Here, we report the complete chloroplast genome of *S. tricarpum*. It shows a typical quadripartite structure with a total length of 149,349 bp, including the large single-copy region (LSC) of 81,644 bp, the small single-copy region (SSC) of 16,643 bp, and two separated inverted regions (IRs) of 25,531 bp, respectively. For the whole genome, there are a total of 131 genes, including 85 protein-coding genes (PCGs), 8 rRNA genes, and 37 tRNA genes. The overall GC content of the cp genome is 37.8%. A well-supported phylogenetic tree revealed monophyly formed by *S. tricarpum* and *S. sarmentosum*, suggesting a relatively closer phylogenetic relationship with the clade consisting of *S. lineare* and *Graptopetalum amethystinum.* The complete chloroplast genome of *S. tricarpum* provides valuable information for further phylogenetic reconstruction of the Crassulaceae family.

*Sedum tricarpum* Makino., a perennial succulent herb, belongs to the family of Crassulaceae. It was first found to occur in Anhui Province, China and established as a new species (Hong et al. 2013). It normally grows on rocks along small streams at an altitude of about 200–400 m. In the current study, we report the first complete chloroplast (cp) genome of *S. tricarpum* in order to provide valuable information for further phylogenetic reconstruction of the Crassulaceae family.

One individual was collected in Shunxin, Lin’an, Zhejiang Province (23.08°, 113.22°). A specimen was deposited at the herbarium of College of Life Sciences, Zhejiang Chinese Medical University (Jing Chen; cj00123@zcmu.edu.cn) under the voucher number 20150614-050-031). Tender leaves were collected and instantly put into the silica gel for drying and preservation. Total genomic DNA was extracted using a modified CTAB method (Doyle and Doyle 1987). The paired-end (2 × 150 bp) library was sequenced by Illumina PE150 at Novogene Co. Ltd (Beijing, China). A total of 2.34 Gb clean reads were obtained after removing low-quality reads and adaptor sequences., We assembled the complete cp genome of *S. tricarpum* using GetOrganelle (Jin et al. 2020) and SPAdes (Bankevich et al. 2012). Manual adjustment and annotation were conducted with the aid of Geneious prime 2019.2.1 (Kearse et al. 2012). The tRNA genes were annotated with ARAGORN (Laslett and Canback 2004). The cp genomes of 13 species of Crassulaceae were downloaded from the GenBank database, including *Aeonium arboreum* (MW206792), *Crassula perforata* (MW206794), *Graptopetalum amethystinum* (MW206795), *Hylotelephium ewersii* (MN794014), *Kalanchoe daigremontiana* (MT954417), *Phedimus aizoon* (MN794321), *Phedimus takesimensis* (KF954541), *Rhodiola sacra* (MN109978), *Sedum lineare* (MT755626), *Sedum sarmentosum* (JX427551), *Sinocrassula densirosulata* (MW206800), *Sinocrassula indica* (MN794334), and *Umbilicus rupestris* (MN794335). *Hamamelis mollis* (MH191387) of Hamamelidaceae was used as an outgroup. A total of 14 cp genomes were aligned with online software MAFFT on CIPRES (https://www.phylo.org, Miller et al. 2015). Maximum likelihood (ML) analyses were performed using RAxML-HPC v.8.2.10 on XSEDE (https://www.phylo.org) with 1,000 bootstrap replicates. Substitution model of GTR + I + G (Stamatakis 2014) was applied which was determined by the Bayesian information criterion (BIC) in jModeltest v2.1.10 (Darriba et al. 2012).

The *S. tricarpum* cp genome has been deposited in GenBank (Accession No.: MZ519883). The total length is 149,349 bp with the typical quadripartite structure. It consists of a pair of inverted regions (IRs) of 25,531 bp separated by a large single-copy region (LSC) of 81,644 bp and a small single-copy region (SSC) of 16,643 bp, respectively. The overall GC content of the cp genome is 37.8%. The whole cp genome of *S. tricarpum* contains 131 genes with 85 protein-coding genes (PCGs), 37 tRNA genes, and eight rRNA genes. Among these genes, 63 PCGs and 22 tRNA genes are located in the LSC region, while 12 PCGs and one tRNA gene occur in the SSC region. All of the eight rRNA genes are duplicated in the IR regions. Additionally, IR regions contain five PCGs and seven tRNA genes, if counting only once. Among the annotated genes, 15 genes (*trnK-UUU, trnL-UAA, rps16, trnV-UAC, rpl2, ndhB, trnG-UCC, trnI-GAU, trnA-UGC, atpF, ndhA, rpoC1, petD, petB, and rpl16*) contain one intron, while three genes (*clpP, ycf3,* and *rps12*) possess two introns. A well-supported phylogenetic tree based on ML analysis was reconstructed, suggesting monophyly formed by *S. tricarpum* and *S. sarmentosum* (Figure 1). The two species demonstrate a comparably closer phylogenetic relationship with the clade that consists of *S. lineare* and *Graptopetalum amethystinum.*

**Figure 1.**
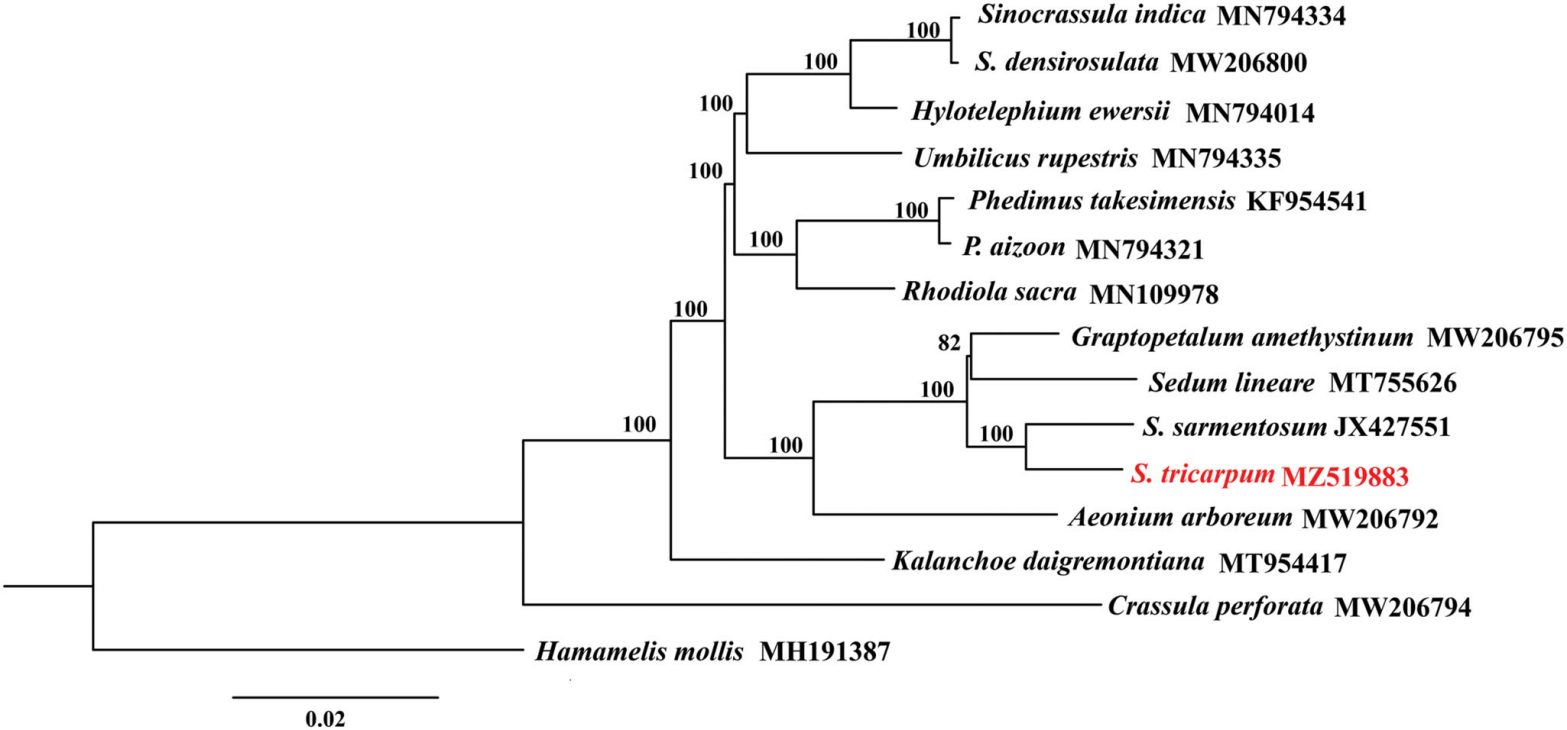
ML tree based on 15 complete chloroplast genomes of Crassulaceae and one outgroup species. Numbers at the nodes are bootstrap support values based on 1000 replicates. GenBank accession numbers are listed beside the species. The species *S. tricarpum* is highlighted in red.

## Data Availability

The complete chloroplast genome sequence data that support the findings of this study are openly available in GenBank of NCBI at (https://www.ncbi.nlm.nih.gov/) under the accession no. MZ519883. The associated BioProject, SRA, and Bio-Sample numbers are PRJNA749476, SRR15239711, and SAMN20371514 respectively.
